# Investigating the effectiveness of protection motivation theory in predicting behaviors relating to natural disasters, in the households of southern Iran

**DOI:** 10.3389/fpubh.2023.1201195

**Published:** 2023-09-07

**Authors:** Reza Faryabi, Fatemeh Rezabeigi Davarani, Salman Daneshi, Declan Patrick Moran

**Affiliations:** ^1^School of Public Health, Jiroft University of Medical Sciences, Jiroft, Iran; ^2^Social Determinants of Health Research Center, Institute for Futures Studies in Health, Kerman University of Medical Sciences, Kerman, Iran; ^3^School of Public Health, University College Cork, Cork, Ireland

**Keywords:** natural disasters, protection, motivation, theory, household, predicting, behaviors

## Abstract

**Background:**

Disasters can lead to large human casualties, destruction of property and economic and environmental resources. The purpose of the present study was to answer the question whether the Protection Motivation Theory (PMT) is effective in predicting behaviors related to the harmful effects of natural disasters in the households of southern Iran.

**Materials and methods:**

This quantitative and cross-sectional study was conducted on 528 households in Jiroft city. Sampling was done by combined method (the combination of cluster, simple random, proportional and systematic random sampling). A total of 528 households were included. The tools for collecting data were demographic information and a researcher-made questionnaire related to PMT constructs and preventive behaviors from the harmful effects of natural disasters. Data was analyzed using SPSS v21 software, and the necessary analyzes (descriptive tests, chi-square, one-way ANOVA and Pearson’s correlation test) were performed at a significance level of 0.05. Using Amos v 21 software, the predictors of safety behaviors were determined using path analysis.

**Results:**

The results showed 51.7% lived in the city and 62.1% of residential buildings were of brick without markings. There is a significant difference between preventive behaviors and direct exposure to disasters (*p* < 0.001), education (*p* = 0.004), monthly income (*p* = 0.004) and source of information (*p* = 0.040). There was also a significant correlation between preventive behaviors and the number of vulnerable family members (*p* = 0.001, *r* = 0.160). The adjusted model of the path analysis test showed that protection motivation (β = 0.547), fear (β = 0.147) and perceived vulnerability (β = 0.135) had the greatest role among the constructs of the protection motivation theory.

**Conclusion:**

According to the results of the study, it is suggested that health planners design and implement educational interventions based on the structures of the mentioned model to increase the preparedness of households against natural disasters.

## 1. Introduction

A disaster is defined as the severe destruction of systems in society or the severe destruction of the functioning of a society. Disasters can lead to large numbers of human fatalities, loss of property and economic and environmental resources, so that society does not have the ability to provide the necessary medical and non-medical resources to deal with its risks (1). An important point is the amount of fatalities caused by disasters in underdeveloped countries, which is about 43 times that of developed countries (2). The data of the International Federation of Red Cross shows five global crises in the world during the years 2005–2014, which are, respectively, floods, storms, waves, heat and droughts. Of all these natural hazards, 48% happened in Asia. Building flexibility and minimizing losses caused by natural hazards are among the most important priorities of all governments in the world, which require the investment of governments and people (3).

Natural disasters cause loss and damage and may influence subjective expectations about the incidence and severity of future disasters. These expectations may in turn shape people’s behaviors in the face of disasters, potentially spending their incomes on disaster risk prevention and mitigation (4). As a result, considering the global changes in climate, weather and environmental changes, it is necessary to think of measures to reduce the risk of natural disasters. During the past two decades, decision-making in natural disaster risk management has progressed significantly. This has led to a refocusing from the modern top-down, “command and control” approach to disaster management to encouraging “people-centered” methods and local participation (5).

Because effective preparation of individuals and families is needed before interventions can be implemented to deal with the effects of a disaster, it is important to understand why people do or do not act on disaster preparedness (6). Therefore, it is necessary to measure people’s risk perception and behavior related to natural disasters in order to reduce the public health problems caused by these disasters (destruction of infrastructure, deaths, physical and mental illnesses, and disability) as much as possible (7). Even expanding the capabilities of disabled people is critical to deal with disaster. This can be considered by strengthening the meaningful participation of disabled people in decision-making processes on their well-being, not only during natural disasters, but also in everyday life (8).

Protection Motivation Theory (PMT) as a health promotion model states that a degree of risk-related information can create the necessary motivation to determine the severity of risk, vulnerability and ability to reduce risk in people (6). PMT has become a popular theory to explain residents’ risk reduction behavior against natural hazards. This theory includes two stages of threat appraisal (perceived vulnerability, perceived severity, and reward) and coping appraisal (response efficacy, self-efficacy and response costs) and the construct of fear (9). According to this theory, a person is likely to perform preventive behaviors if they believe there is a possibility of a risk occurring (perceived vulnerability) and the consequences of the risk are serious (perceived severity) and in addition, the perceived internal and external rewards are less than existing behaviors that increase the probability of harm. Also, perceived self-efficacy and perceived response efficacy should overcome adaptive response costs. Protection motivation is synonymous with the behavioral intention that causes or continues the protective behavior (10).

In Wunnava’s study, regression results showed self-efficacy and response costs were significant and consistent predictors of disaster recovery planning (11). The results of Tang’s studies showed that self-efficacy, response efficacy, and perceived costs were significantly correlated with protection motivation and disaster preparedness behavior (6).

In addition to thanking you for your valuable comment, considering that in the investigated region, various natural disasters such as drought, floods, earthquakes and storms threaten the people of the region and there are common behavioral factors in them (for example, construction of safe housing and retrofitting of buildings). Can increase the safety of households against most natural disasters and individual natural disasters have been addressed in numerous researches, the innovation of our study is that it addressed the common behavioral factors of natural disasters with the protection motivation theory. Considering the importance of preventive measures related to reducing the harmful effects of natural disasters and the results of the studies mentioned above and significant occurrence of various natural disasters such as floods, earthquakes, droughts, storms, etc. in the Jiroft city, south of Iran, and the different cultural, economic-social and climatic conditions of southern Iran compared to other regions of the country and other countries, and so far that do not study to examine the preparedness of households in southern of Iran against natural disasters, it was decided to conduct this study titled “Investigating the Effectiveness of PMT in Predicting Behaviors Relating to Natural Disasters, in the Households of Southern Iran.”

## 2. Methods

This was a cross-sectional study with a descriptive-analytical approach, which was conducted with the aim of determining the effectiveness of PMT in predicting the behaviors of coping with the harmful effects of natural disasters in the households in the south of Iran in 2022. The studied community included all the households in Jiroft city, south of Iran, who had their own residential house. Sampling was done by a combined method (cluster, simple random, proportional and systematic random sampling), so that each of the urban and rural healthcare centers of Jiroft city is considered as a cluster, 3 urban centers and 3 rural centers were selected by a simple random method, health centers, rural health houses and urban health posts were randomly selected (in total, 7 rural health house and 3 urban health post were included in the study). In each health house and health post, according to the total number of households, a number of households were included in the study according to the list of households and randomly based on the number of households in each health center.

A total of 570 people were included in the study, 42 questionnaires were incomplete, and therefore, the data of 528 people (273 urban households and 255 rural households) were analyzed. In each household, questions were asked from one person who met the entry criteria. The inclusion criteria included being native and resident of the region, owning a private home, and being able to understand and answer the questions in the questionnaire. Questions related to the literacy and comprehension skills of the head of the household, or his wife. Exclusion criteria included being non-native and non-resident of the area, having temporary housing (living in a tent) and renting, not being able to speak clearly and answer questionnaire questions. Next, utilizing trained personnel, demographic information and a questionnaire created by the researcher a survey was completed by visiting the homes of the selected households where consent to participate in the study was given. Before completing the questionnaires, the objectives of the study were explained to the selected households by trained personnel and they were assured that their information would remain confidential, and if they had informed and freely verbal consent to participate in the study, the questionnaires were completed. In cases where we could not visit the households, questions were asked to the head of the family or his wife by phone after they gave free and informed consent to participate in the study. If a household did not meet the required conditions or was not willing to participate, a replacement was randomly selected from the unselected households.

The researcher-made questionnaire consisted of three parts. The first part of 15 demographic questions (including age, gender, residence status, education, occupation, life status with spouse, monthly income, type of building, number of years since the construction of the house, type of house ownership, number of family members, number of vulnerable people in family, height of the building from the ground, direct exposure to disasters, source of information for disaster management), the second part of the questionnaire related to the constructs of PMT, which generally consists of 46 questions, including 9 questions of perceived severity, There are 6 perceived vulnerability questions, 5 perceived costs questions, 4 perceived rewards questions, 9 self-efficacy questions, 7 response efficacy questions, 5 fear questions, and one protection motivation question. All questions of the protection motivation theory had a 5-point Likert response. The questionnaire of PMT was scored between 46 and 230. The third part included 27 questions about the behaviors related to reducing the harmful effects of natural disasters with yes and no options, the yes option was given a score of 2 and the no option was given a score of 1. The total score of the behaviors section was between 27 and 54. The validity of the questionnaire was confirmed according to the opinion of a panel of judges by measuring CVR and CVI indices and its reliability was confirmed through the completion of the questionnaire by 31 people from the target community using the test–retest method. Finally, after collecting the information, the data was entered into the SPSS v21 software, and the necessary analysis was performed using descriptive tests (number and percentage, mean and standard deviation), chi-square, one-way ANOVA and Pearson’s correlation test at the mean level 0.05. Using Amos v 21 software, the predictors of safety behaviors were determined using path analysis.

## 3. Results

The results of this study showed 45.8% of the participants were less than 40 years old, 51.7% lived in the city, and 62.1% of the residential buildings were of ordinary brick type (Table 1).

**Table 1:** Demographic and building characteristics of study participants (*n* = 528).

Variable		Number	Percent	Variable		Number	Percent
Age	Less than 40	242	45.8	Years pass to building	Less than 3 year	66	12.5
	40–60	153	29		3–10 year	130	24.6
	Above 60	133	25.2		10–20	173	32.8
Residual status	City	273	51.7		20–30	117	22.2
	Rural	255	48.3		Above 30 year	42	8
Sex	Male	126	23.9	Ownership status	Personal	358	67.8
	Female	402	76.1		Father’s house	141	26.7
Literacy	Elementary	136	25.8		Children’s house	29	5.5
	Guidance to diploma	296	56.1	Number of family persons	One person	16	3
	University	96	18.2		Two person	128	24.2
Job	Farmer/worker	124	23.5		3–5 person	285	54
	Employee	161	30.5		6 person and above	99	18.8
	Retirement	83	15.7	Number of vulnerable person in household	0	167	31.6
	Housewife	117	22.2		1 person	108	20.5
	Other	43	8.1		2 person	143	27.1
Living situation with spouse	Living with spouse	481	91.1		3 person and above	110	20.8
	Divorced or death spouse	47	8.9	The height of building from the ground	Good	270	51.1
Monthly income	Less than 400 dollars	351	66.5		Bad	258	48.9
	400–800 dollars	147	27.8	Direct exposure to disorders	Yes	119	22.5
	Above 800 dollars	30	5.7		No	409	77.5
Types of building	clay and mud	40	7.6	Source of information	TV/radio	163	30.9
	Brick without markings	328	62.1		Social networks	103	19.5
	A brick with markings	96	18.2		Health workers	138	26.1
	Steel-framed building	38	7.2		Internet	79	15
	Concrete structure building	26	4.9		Other	45	8.5

In terms of the percentage of score obtained from the total score that can be obtained for the constructs of PMT and protective behaviors, the constructs of response costs and response efficiency scored the highest and preventive behaviors scored the lowest (Table 2).

**Table 2:** The average score of PMT constructs and behaviors related to coping with disasters in the households participating in the study.

Variable	Mean	SD	Minimum	Maximum	Attainable score	Percent’s of attained score
Perceived vulnerability	19.84	6.04	6	30	6–30	66.13
Perceived severity	31.45	6.49	15	45	9–45	69.88
Perceived rewards	13.8	4.34	4	20	4–20	69
Perceived costs	19.6	4.44	5	25	5–25	76.64
Self-efficacy	31.28	6.00	10	43	9–45	69.51
Response efficacy	26.34	5.58	8	35	7–35	75.25
Fear	17.96	5.55	5	25	5–25	71.84
Protection motivation	3.60	1.42	1	5	1–5	72
Safety behaviors	31.22	2.40	27	38	27.54	57.81

Using chi-square test, there was a significant difference between preventive behaviors and direct exposure to disasters (*p* < 0.001). Using one-way analysis of variance, there was a significant difference between preventive behaviors and literacy level (*p* = 0.004), monthly income (*p* = 0.004) and source of information (*p* = 0.040). Using Pearson’s correlation test, there was a significant correlation between preventive behaviors and the number of vulnerable people in the family (*p* = 0.001, *r* = 0.160).

In terms of the correlation between the protection motivation theory constructs and preventive behaviors from the harmful effects of natural disasters, there is an inverse and significant relationship (*p* < 0.05) between the constructs of perceived rewards and perceived costs and a direct and significant relationship between other PMT constructs and preventive behaviors (*p* < 0.01) was found (Table 3).

**Table 3:** Correlation between the constructs of PMT and natural disaster coping behaviors (Pearson correlation test).

Variables	Perceived vulnerability	Perceived severity	Perceived rewards	Perceived costs	Self-efficacy	Response efficacy	Fear	Protection motivation	Safety behaviors
Perceived vulnerability	1								
Perceived severity	0.254**	1							
Perceived rewards	0.015	−0.045	1						
Perceived costs	−081	−0.063	−0.121**	1					
Self-efficacy	0.071	0.000	−0.053	−0.004	1				
Response efficacy	0.158**	0.104*	−0.075	−0.125**	0.204**	1			
Fear	0.262**	0.414**	−0.036	−0.041	0.117**	0.103*	1		
Protection motivation	0.126**	0.283**	−0140**	−0.139**	0.247**	0.202**	0.424**	**1**	
Safety behaviors	0.166**	0.174**	−0.089*	−0.086*	0.166**	0.115**	0.121**	0.503**	**1**

*p-value < 0.05; **p-value < 0.01.

According to Table 4, the constructs of perceived vulnerability, fear and protection motivation had a significant direct effect among all the constructs of the theory of protection motivation to predict protection behaviors against the effects of natural disasters.

**Table 4:** Direct and indirect effects of variables on safety behaviors.

Variables	Standardized direct effects	Standardized indirect effects	Standardized total effects
Perceived vulnerability	0.135	0.006	0.141
Perceived severity	–	0.082	0.082
Response costs	–	−0.051	−0.051
Perceived rewards	–	−0.052	−0.052
Fear	−0.147	0.183	0.036
Self-efficacy	–	0.104	0.104
Response efficacy	–	0.054	0.054
Protection motivation	0.547	–	0.547

According to Table 5, the statistical indicators of the adjusted model show a reasonable adjustment.

**Table 5:** Statistical indicators of adjusted model.

RMSEA^a^	NFI^b^	CFI^c^	GFI^d^	AGFI^e^	CMIN	DF^f^	CMIN/DF^g^	*p*-value	χ^2^
0.000	0.992	1	0.998	0.990	4.705	9	0.523	0.859	4.705

a Root mean square error of approximation (RMSEA).

b The normed fit index (NFI).

c The comparative fit index (CFI).

d The goodness of fit index (GFI).

e Adjusted Goodness of Fit Index.

f Degrees of freedom (DF).

g Minimum discrepancy per degree of freedom (CMIN/DF).

**Figure 1:**
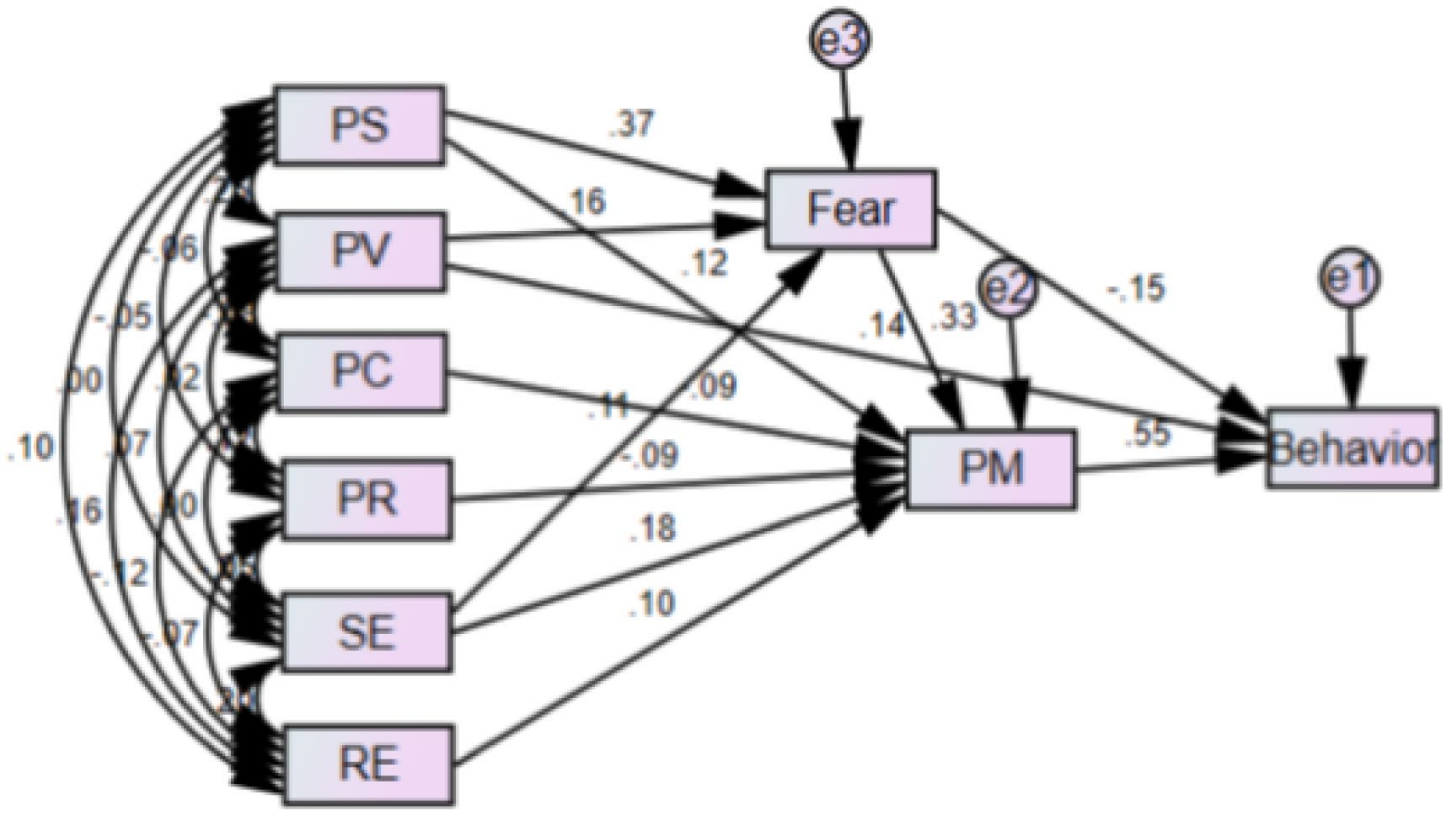
Modified model for safe behavior of households related to hazardous effects of the natural disasters (In this shape, PV = perceived vulnerability, SE = self-efficacy, RC = response costs, PR = perceived rewards, PS = perceived severity, PM = protection motivation).

Figure 1 shows that the PMT constructs predict 85.9% of the variance in safety behaviors. Among these constructs, protection motivation (β = 0.547), fear (β = −0.147), and perceived vulnerability (β = 0.135) had more significant roles than the others.

Therefore, the model presented in Figure 1 can be a suitable model for predicting protective behaviors against the harmful effects of natural disasters.

## 4. Discussion

The purpose of the present study was to answer the question whether the Protection Motivation Theory (PMT) is effective in predicting behaviors related to the harmful effects of natural disasters in the households of southern Iran. Based on the results of this study, preventive behaviors scored the lowest in the investigated households. In other studies conducted in various locations throughout the world, although there are differences in the level of preventive behaviors against risks in different societies, the results show that most households do not take preventive measures and do not have the necessary preparation against risks (12–14), for example, in the study of Han et al. (15) in China, only 44% of households stated that they had taken protective measures against earthquakes after the 2008 earthquake. Also, despite the implementation of educational programs by the responsible organizations in Iran, the results of most of the conducted studies indicate the inadequacy of preventive measures and preparedness of Iranian households against risks, and the households have shown relatively high vulnerability to potential risks (16). Studies have shown that various factors such as quality of life, trust in authorities and the government, psychological factors such as fear and stress, socio-economic factors, knowledge level, self-efficacy, health status and experience of disasters affect preventive behaviors and household preparedness against impact of hazards (17–22). Therefore, identifying obstacles and inhibiting factors and designing appropriate interventions can improve preventive behaviors in communities.

Based on the results of this study, in the studied households, the structure of response costs got the highest score, which means preventive behaviors have many costs (financially, equipment, time consuming, etc.) for households. Households perform preventive behaviors and prepare for risks if they are sure that they have the ability to overcome the costs of the recommended actions. According to the results of qualitative studies conducted by Rezabeigi Davarani et al. (17) in Kerman and Khosravi et al. (23) in Kermanshah, almost all the participants stated financial issues as a major obstacle for preventive measures and preparing households against earthquakes.

The results of the present study showed there is a significant difference between preventive behaviors and direct exposure to disasters. In a study conducted by Greer et al. (24) in the United States, earthquake experience was a significant predictor of risk reduction behaviors and in a study by Han et al. (15) in China, earthquake experience and concern for future damage had a significant effect on preventive behaviors and preparedness against earthquakes. In the study of Ansari et al. (25), flood experience had an effect on the protection motivation in flood-prone households.

Suffering from injuries and damages caused by destructive risks can make the victims take more protective and preventive measures than households that did not experience destructive risks. In Sun and Xue’s (26) study in China, the relationship between the experience of a non-destructive earthquake and the intention to prepare against an earthquake was not statistically significant. Populations who have experienced non-destructive risks may consider the occurrence of frequent and non-destructive risks to be normal for them, and a false sense of security prevents them from taking preventive measures and preparing for risks. Also, studies have shown that the type of risk experienced can have an impact on the preventive measures of communities. For example, the results of Wei et al.’s (14) study in Taiwan showed that people with hurricane experience were significantly more prepared against hurricanes, while earthquake experience was not significantly related to the level of preparedness. Due to the unpredictability of the exact time and place of an earthquake, people may feel that they have less control over the risk of an earthquake and take less preventive measures.

In the present study, there was a significant difference between preventive behaviors and education. The results of other studies conducted in different parts of the world showed those with a higher level of education, took more preventive measures and had higher preparation against risks (12, 13, 20, 27–29). Considering that the educated class may have better economic conditions, they are more able to perform protective and preventive measures, especially strengthening and purchasing equipment. For example, the results of the study by Twerefou et al. (30) in Ghana showed that socio-economic factors have an overall positive effect on protective behavior against floods.

In this study, there was a significant difference between preventive behaviors and monthly income. In other studies conducted, including the study of Inal et al. (28) in Turkey, Kelly and Ronan (31) in New Zealand, Tang and Feng (6) in Taiwan, Wang et al. (27) in China, Armaş et al. (32) in Romania, and Zakour and Kim (33) in United States. The higher the household’s job position and income, the more preventive behaviors and preparedness measures they reported against risks. One of the most important basic measures to reduce the vulnerability of households to hazards is the construction of standard and resistant houses. Buying land in low-risk areas and building a durable and standard house requires a lot of financial resources, and low-income households may not be able to pay the high costs of retrofitting their houses and buying equipment for emergency situations.

The results of this study showed that there was a significant correlation between preventive behaviors and the number of vulnerable people in the family. In a study by Han et al. (34) in Taiwan, having a disabled member in the family was not a significant predictor of adopting emergency preventive measures, and households with a disabled member were less prepared for natural hazards. Perhaps in families where there is a vulnerable person, due to the need for continuous care, caregivers do not have enough time to participate in training classes and exercises. In a study by Adams et al. (18) that was conducted on disabled individuals over 18 years old, the results showed that participants who were in poor health and had activity limitations were less involved in preventive behaviors and disaster preparedness. Therefore, it is necessary to design and implement special interventions for vulnerable groups and other family members to promote preventive behaviors for households that have a vulnerable member at home.

In the present study, an inverse and significant relationship was observed between behaviors that prevent the harmful effects of natural disasters and the perceived rewards and costs constructs. In the study of Greer et al. (24) in the United States and Adhikari et al. (35) in Nepal, perceived costs were associated with household preventive actions. In the Cong study in the United States, perceived cost was a barrier to household disaster preparedness (36). In Botzen’s study in New York, perceived cost did not play a role in flood damage reduction measures (37). In Sun and Xue’s (26) study in China, residents had a lower intention to purchase earthquake insurance and stockpile equipment for emergencies than to participate in training and exercise activities. Households are likely to take actions such as participating in training and training courses that do not require a lot of financial resources, rather than expensive actions such as buying insurance and providing equipment.

In this study, a direct and significant relationship was found between the constructs of perceived vulnerability, perceived severity, self-efficacy, response efficiency, and fear and protection motivation with preventive behaviors. In the study of Greer et al. (24), the likelihood of an earthquake and the perceived consequences, self-efficacy and response efficacy were significantly related to the preventive actions of households. In a study conducted by Adams et al. (18) in the United States, self-efficacy and response efficacy were significantly positively related to preventive behaviors and disaster preparedness. The results of Adhikari et al.’s (35) study in Nepal showed perceived vulnerability, self-efficacy and response efficacy predicted the intention to prepare against risks and the relationship between these constructs with the intention to prepare behavior was significant. In Botzen’s study in New York, high response efficacy and high self-efficacy played an important role in flood damage reduction measures (37).

The results of a systematic review study showed that people with higher self-efficacy took more preventive measures against disasters and had more preparedness intentions and behavior (38). In the Cong study in the United States, low self-efficacy and response efficiency were reported as barriers to household preparedness against disasters (36). People are likely to perform preventive and preparedness behaviors against disasters if they are confident the actions they take to prepare will reduce the consequences of the hazard and the probability of harm (response efficacy) and they are confident of their ability to perform preparedness and preventive behaviors (self-efficacy). The results of this study show the more motivated people are to take preventive measures against risks, the more preventive and protective behavior increases within them. In fact, the motivation to protect is synonymous with the behavioral intention that triggers or continues the protective behavior (10). In a study conducted by Zaremohzzabieh et al. (39) in Malaysia, the intention to perform behavior was directly related to preventive measures and preparedness of households against earthquakes.

In this study, fear was one of the predictors of preventive behaviors. Studies have shown that the feeling of fear and worry about the consequences of risks has an affect on preventive behaviors. For example, in Stewart’s (40) study in the United States, fear of extreme consequences of climate hazards predicted the likelihood of residents evacuating. In the study by Ao et al. (12) in China, negative emotions (being nervous, afraid, and anxious) during an earthquake had a positive effect on preventive measures and preparedness of households against earthquakes. The study by Ansari et al. (25) showed fear of flooding has a positive correlation with risk reduction measures. In Greer et al.’s (41) study in the United States, feelings of fear or negative emotions related to earthquakes were significant predictors of intention to take risk reduction measures. In fact, the fear of the consequences of risks can make people adopt strategies to deal with the risk, therefore, it is necessary to design and implement the necessary training and sensitizing the society regarding the consequences of risks through different channels.

In this study, perceived vulnerability was one of the important predictors of preventive behaviors. The results of Ong et al.’s (42) study in the Philippines showed that the higher the perceived vulnerability of people, the greater the intention to prepare against earthquakes. Kurata et al. (43) showed that perceived vulnerability has a direct positive effect on evacuation intentions, beliefs and preparedness behaviors of Filipinos against the risk of volcanic eruption.

Finally, according to the results of the present study and the theoretical framework presented in this research, the more people see themselves exposed to risks and are aware of their vulnerability (high perceived vulnerability to the occurrence of natural disasters) and their fear of undesirable natural disasters, their motivation to protect themselves and others (protection motivation) is greater to take preventive and protective measures against the destructive effects of natural disasters. Therefore, carrying out appropriate health education and health promotion interventions can increase households’ understanding of their vulnerability to natural disasters and, consequently, be effective in carrying out effective preventive measures against the effects of undesirable natural disasters.

## 5. Limitations

One of the limitations of this study is the prolongation of the data collection period due to the spread of COVID-19 in the region.

## 6. Conclusion

Based on the results of this study, PMT was a suitable scientific framework for predicting preventive behaviors against the potentially dangerous risks of disasters, the higher the perceived vulnerability, perceived severity, self-efficacy, response efficiency, fear and protection motivation, and the lower the rewards and cost of the perceived response, the more people take preventive measures. It seems that PMT can be used in developing educational programs and intervention techniques in order to increase preventive measures. In this study, preventive behaviors of households against risks were not favorable, one of the reasons for this is the high cost of protective and preventive measures and the insufficient income of most households. Therefore, the support of the authorities is necessary to reduce the vulnerability of the society. Considering the lower level of preventive behaviors in people with low education level, appropriate educational interventions should be prioritized in order to increase the knowledge level of all sections of the society, especially vulnerable groups. Considering the source of information for most of the participants was mass communication media and health workers, with correct and appropriate planning, the great potential of media and health workers can be used to transmit appropriate messages in order to increase awareness and improve preventive and protectively behaviors. It is suggested to conduct interventional studies to measure the amount of PMT-based interventions on preparedness behaviors to reduce the harmful effects of natural disasters.

## Data availability statement

The original contributions presented in the study are included in the article/supplementary material, further inquiries can be directed to the corresponding author.

## Ethics statement

The studies involving humans were approved by this study is approved by the ethical committee of Jiroft University of Medical Sciences with the number of IR.JMU.REC1400.034, all the methods were performed in accordance with the relevant guidelines and regulation. The studies were conducted in accordance with the local legislation and institutional requirements. The participants provided their written informed consent to participate in this study.

## Author contributions

RF, SD, and FR were involved in all aspects of study conception and design, data collection, data analysis, interpretation, drafting of the manuscript, and critically revising the manuscript for intellectually important content. DM helped in the general design of the study, results from interpretation, co-authoring, and editing along with the whole manuscript. All the authors have read and approved the final version of the manuscript and agreed to be accountable for all aspects of the work.

## Conflict of interest

The authors declare that the research was conducted in the absence of any commercial or financial relationships that could be construed as a potential conflict of interest.

## Publisher’s note

All claims expressed in this article are solely those of the authors and do not necessarily represent those of their affiliated organizations, or those of the publisher, the editors and the reviewers. Any product that may be evaluated in this article, or claim that may be made by its manufacturer, is not guaranteed or endorsed by the publisher.
